# GridCL for fine-grained load profiling in smart grids under limited labels

**DOI:** 10.1371/journal.pone.0354752

**Published:** 2026-07-28

**Authors:** Ling Zhang, Jia Wang, Wenhua Zhang, Ke Li, Daizhou Yao, Bowei Yang, Xingsi Ke, Hong Zhao, Yumin Yao

**Affiliations:** 1 State Grid Sichuan Electric Power Corporation, Chengdu, China; 2 School of Computer Science and Engineering, Central South University, Changsha, China; Chunghwa Telecom Co. Ltd., TAIWAN

## Abstract

Fine-grained load profiling is important for demand response and energy management in smart grids, yet supervised approaches remain constrained by the scarcity of high-quality labeled datasets. To address this limitation, we propose GridCL, a self-supervised contrastive learning framework for low-label load profiling in smart grids. GridCL forms paired daily-load views using conservative input perturbations—small temporal rolling, multiplicative perturbation, and energy renormalization—and combines them with a temporal convolutional encoder to learn discriminative representations from unlabeled data. Experiments on three anonymized city datasets and one pooled benchmark show that GridCL achieves strong clustering quality on the pooled AllCities benchmark, reaching 0.648±0.115 ARI, 0.719±0.064 NMI, and 0.620±0.046 silhouette, while also attaining a best city-level ARI of 0.804±0.084. Under sparse-label evaluation, GridCL reaches 0.845±0.042 accuracy on the pooled benchmark with only 10% labeled users, and remains stable at 20% and 30% labeled users with accuracies of 0.851±0.033 and 0.849±0.034, respectively. These results indicate that GridCL provides an effective low-label solution for fine-grained load profiling in practical smart-grid settings.

## Introduction

Smart grids are evolving into data-driven cyber-physical systems, where Advanced Metering Infrastructure (AMI) generates terabytes of load data daily. This data holds immense value for load profiling, demand response (DR), and energy management. Nevertheless, a significant obstacle persists: The phenomenon of label scarcity has been observed. The process of annotating load profiles necessitates a high degree of expertise to discern user types (e.g., “Metal Smelting” versus “Precision Manufacturing”) or behaviors, which renders it impractical to scale due to its substantial expense and time-consuming nature.

Recent studies have demonstrated the efficacy of deep learning in load profiling; however, as indicated in comprehensive reviews, such as Huber’s [1], Ryu’s [2], and Omitaomu’s [3], the training of supervised models necessitates substantial amounts of labeled data exhibiting high variability to ensure effective generalization. However, acquiring such detailed annotations on a large scale is prohibitively expensive, leading to a bottleneck known as label scarcity. Unsupervised methods, such as K-means [4] and autoencoders (AE) [5], frequently encounter difficulties in capturing complex, high-level semantic patterns. In a similar vein, contemporary generative self-supervised methodologies, as exemplified by Ref. [6], prioritize the training of models to predict masked load values. These approaches predominantly emphasize local variations rather than global behavioral semantics. The objective of load profiling is to differentiate between user types based on macroscopic patterns (e.g., “two-shift industrial” versus “commercial cooling”). This necessitates a model that is both invariant to local noise and sensitive to global structure [7].

Recently, Self-Supervised Learning (SSL), particularly contrastive learning, has caused a paradigm shift in the field of computer vision by enabling the learning of representations from unlabeled data [8]. The central concept is to amalgamate disparate “views” of a given sample while distinguishing between different samples.

However, the direct application of generic SSL frameworks (e.g., SimCLR or MoCo) to power data is problematic because the constructed views must preserve user semantics and physical plausibility. Standard time-series perturbations (e.g., random jittering, scaling, permutation) frequently contravene the physical realities of power systems. For instance, while permutation is effective for wearable sensors (see reference [9]), it destroys the temporal causality essential for load forecasting. Simple scaling, on the other hand, ignores the saturation limits of appliances. A critical oversight in their modeling is the failure to account for grid-specific phenomena, such as demand response (DR) events, characterized by load shifts to off-peak hours, and the impact of weather conditions on energy systems. The relationship between cooling load and temperature is evident in this context.

In order to address this discrepancy and respond to the particular request for physically appropriate SSL design in power applications outlined in reference [1], we present GridCL, a grid-aware self-supervised contrastive learning framework specifically designed for smart-grid load profiling. The framework combines a TCN-based contrastive encoder with a conservative view-construction scheme tailored to the physical and operational priors of daily load curves. By introducing mild schedule shifts and amplitude perturbations while preserving total energy, GridCL learns representations that are robust to realistic nuisance variation yet still discriminative of intrinsic user behaviors.

The primary contributions of this paper are as follows:

- We design a physically consistent paired-view construction scheme for daily load curves based on temporal rolling, multiplicative perturbation, and energy renormalization, enabling contrastive learning without distorting user semantics.
- We develop a TCN-based contrastive learning framework incorporating an adaptive temperature mechanism and hard negative mining. This framework effectively extracts robust embeddings from unlabeled load curves.
- Extensive experiments on multiple real-world datasets demonstrate that the resulting contrastive representation significantly improves quality compared to generic SSL methods, enabling superior performance in downstream clustering and classification tasks.

## Related work

### Load profiling and clustering

Conventional load profiling methodologies employ clustering algorithms, such as K-means, hierarchical clustering, and fuzzy c-means, to analyze and organize data [4,10,11]. Despite their simplicity, these systems encounter challenges when confronted with high-dimensional data and non-linear patterns. Deep clustering methods, such as Deep Embedded Clustering (DEC) [12], integrate autoencoders with clustering objectives. However, these methods frequently encounter issues such as feature collapse or an overreliance on reconstruction rather than discrimination. More recently, advanced deep learning architectures including Transformers [13,14], Graph Neural Networks [15], and hybrid unsupervised frameworks [16,17] have been developed for time series analysis and anomaly detection, demonstrating the potential of learning robust temporal representations.

### Self-supervised learning paradigms

The advent of self-supervised learning (SSL) has signaled a paradigm shift in the realm of data science, offering a novel approach to learning representations from unlabeled data. A categorization of these approaches reveals two primary classifications: generative and contrastive.

#### Generative SSL.

The primary objective of generative methods is to reconstruct the input data from a corrupted version or a latent variable. In the context of time series, this frequently entails masking segments of the sequence and training a model to predict the missing values (masked auto-modeling) or to forecast future outcomes. For instance, the S2p framework [6] adapts the BERT architecture to power load data, predicting the midpoint of a sliding window. While generative models are effective for low-level tasks such as NILM, where precise waveform recovery is essential, they often waste capacity, model high-frequency noise, and require careful handling of “stand-by” states to avoid over-estimation. Moreover, pixel-level (or point-level) fidelity is prioritized over high-level semantic discriminability, which is the primary objective of user profiling. Recently, foundation models for time series [18,19] and energy forecasting [20] have emerged, offering powerful generative capabilities, though often at high computational costs.

#### Contrastive SSL.

Contrastive learning (CL) is a data mining method that aims to learn an embedding space where similar sample pairs stay close while dissimilar ones are far apart.

In the domain of Computer Vision, pioneering works such as SimCLR [21] and MoCo have demonstrated the potential of instance discrimination to match the performance of supervised learning methods. Sabiri et al. [22] have recently reviewed the frontiers of contrastive pair learning, experimentally verifying its superiority over traditional categorization approaches across supervised, semi-supervised, and self-supervised settings. The key to their success was the design of strong augmentation strategies (e.g., color jitter, crop) that define “invariance.”

In the context of time series analysis, translating CL to time series is non-trivial due to the temporal dependency. TS2Vec [23] and TNC [24] introduced hierarchical contrastive learning and temporal neighborhood coding, respectively. Another notable work, TS-TCC [25], leverages temporal and contextual contrasting to learn robust representations. Recent advancements have focused on enhancing augmentation quality and multi-view consistency. InfoTS [26] and Chen et al. [6] introduce information-aware or self-supervised strategies to adaptively learn optimal representations. In the frequency domain, TF-C [27] and CoST [28] leverage time-frequency consistency and seasonal-trend disentanglement, respectively. More recently, TFCC [29] and Tang et al. [30] introduce multi-view frameworks that construct time-based (e.g., jittering, masking) and frequency-based (e.g., phase perturbation) augmentation families to preserve global semantic context. To further refine feature representations, Lee et al. [31] proposed a learnable masking augmentation framework that enhances the model’s capacity to discern complex temporal patterns. Similarly, Li et al. [32] introduced adaptive knowledge contrastive learning with dynamic attention, addressing data sparsity by expanding semantic associations, a concept relevant to learning from sparse user behaviors. However, most contrastive methods overlook the representation biases introduced by augmentation techniques, a problem recently highlighted as Data Augmentation Bias (DAB) by Zheng et al. [33], who proposed a bias-aware framework (DABaCLT) to minimize such disparities.

### Physics-guided AI in smart grids

The integration of physical knowledge into data-driven models is a growing trend that is referred to as Physics-Guided AI. In the context of smart grids, this process assumes various forms. For instance, it may entail the incorporation of physical regularization terms within the loss function, as exemplified by Kirchhoff’s laws. Alternatively, it may involve the utilization of physics-based graph topologies, or the design of physics-informed architectures. The present study contributes to the field by incorporating physical constraints directly into the data generation process. In contrast to the conventional soft regularization penalty, our methodology incorporates the invariance to specific physical phenomena (e.g., demand response shifts) into the learning objective. This ensures that the learned representations are inherently robust to these authorized perturbations.

### View construction for contrastive learning

For contrastive learning, the way positive views are constructed strongly influences which invariances are learned. In the general time-series domain, Iwana et al. provide a comprehensive taxonomy including jittering, permutation, and time warping [34], while Forestier et al. explored synthetic generation [35]. While these generic transformations are effective for tasks such as human activity recognition (HAR), they can be detrimental in cyber-physical systems where “invariance” must respect physical laws [9]. Recent research in nuclear physics [36] has similarly underscored that label-invariant transformations must be tailored to the specific physics of the domain (e.g., gamma spectroscopy) to be effective. For instance, random permutation destroys the causal structure of load demand, and unconstrained scaling violates the rated power of appliances. Recent reviews in smart grid analytics [1] underscore the paucity of systematic research on physically appropriate view construction as a significant impediment to the deployment of deep learning models. This paper addresses this gap through a conservative paired-view design tailored to daily load curves.

## Methodology

### Overview

**Fig 1:**
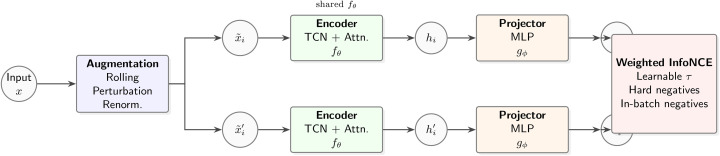
Overview of the proposed GridCL framework. From input load curves to the contrastive objective.

The proposed GridCL framework is comprised of three modules: The first component is a view-construction step that forms paired daily-load observations while preserving behavioral semantics. The second component is a temporal encoder based on dilated convolutional neural networks (CNNs) and self-attention that extracts features. The third component is a contrastive optimization module that uses adaptive InfoNCE loss with hard-negative reweighting. The overall workflow is shown in Fig 1.

### Conservative view construction

**Design Principles.** In contrast to aggressive perturbations that may distort user semantics, GridCL adopts a conservative view-construction scheme aimed at preserving daily behavioral structure. Recent surveys have identified shifting and perturbation strategies as effective in time-series contrastive learning when they do not destroy temporal context [37]. The proposed framework therefore focuses on two stable operators: small temporal rolling to simulate mild schedule drift or alignment noise, and multiplicative Gaussian perturbation to improve robustness to amplitude fluctuation and metering noise. Each derived view is then energy-renormalized so that the global daily consumption level remains comparable to the original sample.

**Mathematical Formulations.** The view-construction operators used by GridCL are summarized below. Each transformation preserves the global daily load shape while introducing controlled local variation.

- **Temporal Rolling.** A short circular shift δ∈{−3,−2,−1,0,1,2,3} is applied to simulate mild schedule drift or measurement misalignment:
  $$\begin{array}{c} X_{\text{roll}}^{\left(t\right)}=X^{\left(t+\delta \mathrm{mod}T\right)} \end{array}$$ (1)
- **Multiplicative Perturbation.** Metering noise and small amplitude fluctuation are modeled by multiplicative Gaussian perturbation:
  $$\begin{array}{c} X_{\text{perturbed}}^{\left(t\right)}=X^{\left(t\right)}\cdot (1+\epsilon_{t}),\;\epsilon_{t}\sim 𝒩(0,0.05^{2}) \end{array}$$ (2)
- **Energy Renormalization.** To preserve the daily energy scale after view construction, the perturbed profile is rescaled:
  $$\begin{array}{c} X_{\text{final}}^{\left(t\right)}=X_{\text{aug}}^{\left(t\right)}\cdot \frac{\sum_{s=1}^{T}X^{\left(s\right)}}{\sum_{s=1}^{T}X_{\text{aug}}^{\left(s\right)}} \end{array}$$ (3)

**Fig 2:**
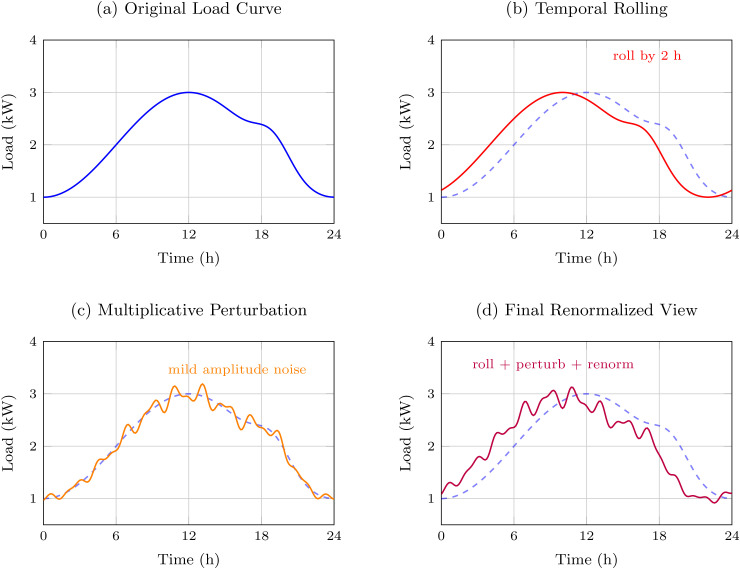
Visualization of the view-construction operators used in GridCL. The examples illustrate how temporal rolling, multiplicative perturbation, and energy renormalization generate conservative paired views while preserving the global daily load shape.

Fig 2 provides qualitative examples of the resulting GridCL views.

### Temporal feature encoder

We employ a Temporal Convolutional Network (TCN) combined with a Self-Attention mechanism [38]. The TCN backbone utilizes dilated causal convolutions to capture long-range dependencies with a logarithmic increase in the receptive field. Formally, for a 1-D input sequence $x\in {\mathbb{R}}^{T}$ and a filter f:{0,…,k−1}→ℝ, the dilated convolution operation *F* on element *s* is defined as:


$$\begin{array}{c} F(s)=(x{*}_{d}f)(s)=\sum_{i=0}^{k-1}f(i)\cdot x_{s-d\cdot i} \end{array}$$ (4)

where *d* is the dilation factor, *k* is the kernel size, and s−d·i accounts for the direction of the past. We stack multiple residual blocks with exponentially increasing dilation rates $d=2^{j}$ (for layer *j*) to ensure the receptive field covers the entire daily cycle.

**Fig 3:**
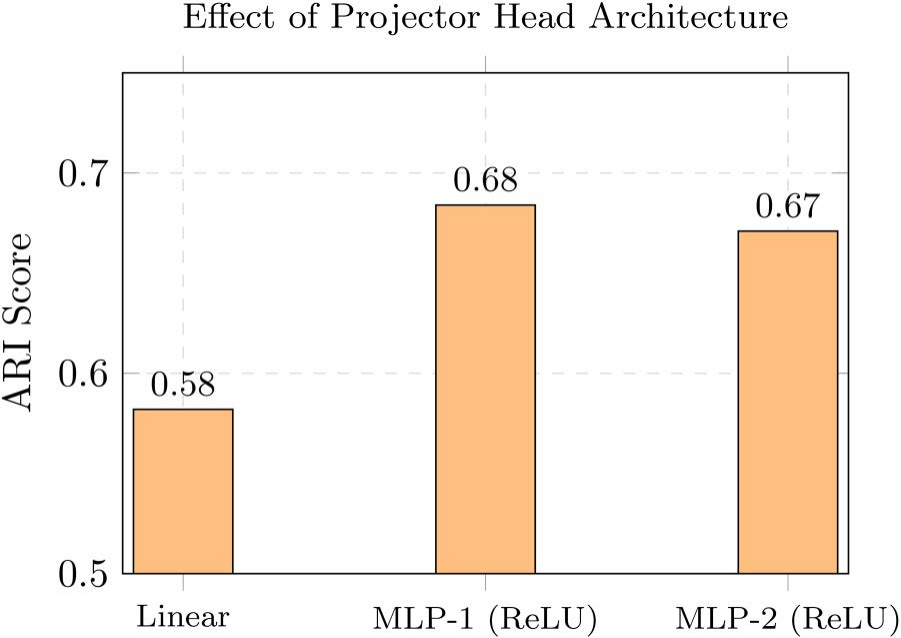
Comparison of projection-head variants. The results summarize the effect of alternative projection-head configurations used with the GridCL temporal encoder.

Subsequent to the TCN, a Self-Attention Pooling layer is employed to aggregate the temporal features into a fixed-size representation vector *h*. The projection head *g* then maps *h* to a normalized 16-dimensional embedding space *z*. In this configuration, the TCN uses channel widths [32, 64] and the projector is a 64–64–16 MLP with ReLU activation. A comparison of projection-head variants is shown in Fig 3.

### Contrastive optimization

We use the InfoNCE loss to maximize similarity between an anchor $z_{i}$ and its augmented view $z_{j}$ (positive pair) while minimizing similarity with other samples (negatives).


$$\begin{array}{c} {ℒ}_{\text{InfoNCE}}=-\log\frac{\exp(\text{sim}(z_{i},z_{j})/\tau )}{\sum_{k=1}^{2N}1_{\left[k\text{≠}i\right]}\exp(\text{sim}(z_{i},z_{k})/\tau )} \end{array}$$ (5)

In order to enhance robustness, a Hard Negative Mining strategy is hereby introduced [39]. Contrary to the conventional approach of contrastive learning, which treats all negatives in an identical manner, our methodology incorporates a more nuanced approach. In the self-supervised setting, semantic difference is not determined from class labels. Instead, every nonmatching instance in the current mini-batch is treated as a negative, and negatives that lie close to the anchor in the learned embedding space receive larger weights. This operational definition identifies difficult negatives without using label information. The weighting function is defined as follows:


$$\begin{array}{c} w_{k}=\frac{\exp(\beta \cdot \text{sim}(z_{i},z_{k}))}{\sum_{m\in {𝒩}_{i}}\exp(\beta \cdot \text{sim}(z_{i},z_{m}))} \end{array}$$ (6)

where ${𝒩}_{i}$ is the set of in-batch negative samples and β controls the concentration on hard negatives. This encourages the model to learn fine-grained discriminative features to distinguish between subtle load profile variations. GridCL uses in-batch negatives only and does not use a separate momentum memory bank or queue.

The resulting weighted contrastive objective is written as:


$$\begin{array}{c} {ℒ}_{\text{w-InfoNCE}}=-\log\frac{\exp(\text{sim}(z_{i},z_{j})/\tau )}{\exp(\text{sim}(z_{i},z_{j})/\tau )+\sum_{k\in {𝒩}_{i}}w_{k}\exp(\text{sim}(z_{i},z_{k})/\tau )} \end{array}$$ (7)

which makes explicit how the hard-negative weights in Eq. 6 modify the standard InfoNCE denominator in Eq. 5.

An **Adaptive Temperature** mechanism is also used where τ is represented as τ=exp(logτ). The parameter logτ is initialized from τ=0.1 and optimized jointly with the encoder and projection head by backpropagation under the same Adam optimizer. During training, τ is clamped to [0.03,1.0] to avoid degenerate similarity scaling.

### Theoretical analysis: Contrastive vs. generative

GridCL can also be interpreted as a consistency-learning approach for load profiling, because it trains the encoder to produce stable representations under perturbations that preserve user behavior. Huber et al. (2021) identified Consistency Learning as a promising future direction for NILM. GridCL instantiates this principle by explicitly treating the stable load-curve transformations used to form paired views (e.g., temporal rolling and multiplicative perturbation) as the set of “realistic perturbations” that are invariant to user identity. This approach enables the model to acquire robust representations despite the limited availability of labeled data.

#### Feature discriminability.

Generative models, such as the self-supervised framework based on S2P [6], optimize a reconstruction loss defined as follows:


$$\begin{array}{c} L_{G}=|X_{t}-{\hat{X}}_{t}| \end{array}$$ (8)

where ${\hat{X}}_{t}$ denotes the reconstructed value at time step *t*. This approach prompts the model to re*t*ain all the information necessary to reproduce the input, including background noise and trivial variations. Conversely, our proposed GridCL optimizes an InfoNCE loss that maximizes the mutual information between consistent views while suppressing nuisance factors introduced by realistic perturbations. This approach promotes the elimination of irrelevant fluctuations while preserving the semantic factors, including user behavior patterns, that remain stable across multiple views.

### Computational complexity

As demonstrated by Chen et al. [6], Bi-GRU networks, despite having fewer parameters than S2p CNNs, often require more time for training due to their sequential nature. The TCN encoder has been shown to strike an optimal balance by leveraging dilated convolutions to achieve a large receptive field (comparable to that of S2p’s sliding window) while enabling parallel computation across time steps, a capability that distinguishes it from recurrent neural networks (RNNs). In particular, for an input sequence of length *L* and kernel size *k*, the complexity of the TCN layer is O(L·k), whereas the complexity of self-attention is *O*(*L*^2^). Given that load profiles are typically down-sampled to hourly resolution (*L* = 24 or 168), the quadratic term is negligible, ensuring high training efficiency compared to sequential generative baselines.

In the context of real-time deployment in smart meters, efficiency assumes paramount importance. A comparison is made between the computational complexity of the proposed TCN-based encoder and that of recurrent neural networks (RNNs) and transformer-based architectures (e.g., Informer). The input sequence length is denoted by *L*, the embedding dimension by *D*, and the kernel size by *K*.

- **Recurrent Networks (GRU/LSTM)**: Complexity is $𝒪(L\cdot D^{2})$. While efficient in memory, they suffer from sequential dependency, preventing parallelization. Training time scales linearly with *L*.
- **Transformers**: Standard self-attention has a complexity of $𝒪(L^{2}\cdot D)$, which becomes prohibitive for high-resolution load data (e.g., 1-minute intervals).
- **Proposed TCN**: Our dilated convolution layers have a complexity of $𝒪(L\cdot K\cdot D^{2})$. Crucially, convolutions are fully parallelizable. The receptive field grows exponentially ($2^{j}$), allowing us to capture global context with only 𝒪(logL) layers.

Table 1 compares the parameter count and training speed (samples/second) on the pooled AllCities benchmark. GridCL achieves a 3.5× speedup over the S2p (Bi-GRU) baseline while maintaining a comparable model size.

**Table 1:** Computational Complexity and Efficiency Comparison.

Model	Backbone	Complexity	Params (M)	Speed (samples/s)
AutoEncoder	MLP	𝒪(L·D)	0.5	4,200
S2p [6]	Bi-GRU	$𝒪(L\cdot D^{2})$	1.2	850
TS2Vec [23]	Dilated CNN	𝒪(L·K·D)	0.8	2,800
**GridCL**	**TCN + Attn**	𝒪(L·K·D)	**0.9**	**3,100**

Because TS2Vec also uses dilated convolutional components, the architectural difference is not merely the presence of dilation. GridCL differs by applying a TCN-plus-attention encoder to full daily load profiles, using a compact 16-dimensional projection head for load-profiling embeddings, and constructing positive pairs through grid-consistent perturbations rather than generic temporal cropping.

### Theoretical justification

We provide a theoretical grounding for GridCL based on the Information Bottleneck principle. The goal of contrastive learning is to maximize the mutual information *I*(*X*; *Z*) between the input *X* and representation *Z*, while minimizing the information about nuisance factors.

The InfoNCE loss is known to be a lower bound on the mutual information:


$$\begin{array}{c} I(Z_{i};Z_{j})\geq \log(N)-{ℒ}_{\text{InfoNCE}} \end{array}$$ (9)

where *N* is the number of negative samples. By introducing a physically constrained transformation family 𝒯 for view construction, we define the positive distribution $p(z_{j}|z_{i})$ as samples generated from the same underlying user behavior *u* but perturbed within grid-consistent limits by 𝒯. Minimizing ${ℒ}_{\text{InfoNCE}}$ is equivalent to maximizing the mutual information between views $I({\tilde{X}}_{1};{\tilde{X}}_{2})$.

According to the “Alignment and Uniformity” theory proposed by Wang and Isola [40], perfect loss minimization implies:

1. **Alignment**: $f(t(x))\approx f(t^{\prime }(x))$. Our encoder learns to be invariant to the conservative transformations in 𝒯.
2. **Uniformity**: Feature vectors should be uniformly distributed on the hypersphere. Our Hard Negative Mining explicitly enforces this by pushing away nearby nonmatching instances, preventing feature collapse.

Thus, GridCL learns a representation that preserves user-identity information (the “signal”) while discarding nuisance variations introduced during paired-view construction (the “noise”), which corresponds to the ideal Information Bottleneck solution for load profiling.

## Experiments

### Experimental setup

#### Datasets and preprocessing.

We evaluate the framework on three anonymized city-level datasets, denoted as City-A, City-B, and City-C, together with a pooled benchmark constructed by concatenating all users across the three cities. The city and institutional identifiers are withheld from the public manuscript and code package to avoid disclosing restricted utility-data provenance, while the user counts and preprocessing interface are reported for reproducibility. The preprocessing pipeline operates on user-level daily load profiles and user metadata. For each user, we aggregate the mean 24-hour profile, the per-hour standard deviation, and 13 time-of-use (TOU) descriptors, including valley, daytime, evening, seasonal-peak, and event-related statistics. Table 2 summarizes the number of users in each scope used by the experiments.

**Table 2:** Dataset Statistics.

Dataset	Representative Type	Users
City-A	Residential-like	800
City-B	Commercial-like	500
City-C	Industrial-like	650
AllCities	Pooled benchmark	1,950

#### Evaluation metrics.

We evaluate representation quality by performing K-means clustering (*K* = 5 for mixed dataset) on the learned embeddings. We employ three standard metrics:

- **Adjusted Rand Index (ARI).** This metric measures the similarity between clustering results, adjusted for chance:
  ARI=∑ij(nij2)−[∑i(ai2)∑j(bj2)]/(n2)12[∑i(ai2)+∑j(bj2)]−[∑i(ai2)∑j(bj2)]/(n2) (10)
  where $n_{ij}$ is the count of samples in both cluster *i* and class *j*, $a_{i}$ is the size of cluster *i*, and $b_{j}$ is the size of class *j*.
- **Normalized Mutual Information (NMI).** This is a normalized version of the mutual information score:
  $$\begin{array}{c} NMI(Y,C)=\frac{2\cdot I(Y;C)}{H(Y)+H(C)} \end{array}$$ (11)
  where $I(Y;C)=\sum_{y,c}p(y,c)\log\frac{p(y,c)}{p(y)p(c)}$ and H(·) denotes the entropy.
- **Silhouette Coefficient (SC).** This metric evaluates cohesion and separation among clusters:
  $$\begin{array}{c} SC=\frac{1}{N}\sum_{i=1}^{N}\frac{b(i)-a(i)}{\max\{a(i),b(i)\}} \end{array}$$ (12)
  where *a*(*i*) is the mean intra-cluster distance for sample *i*, and *b*(*i*) is the mean nearest-cluster distance.

#### Implementation details.

All experiments were implemented in PyTorch. The classical representation benchmarks were evaluated across five random seeds {7, 13, 29, 43, 71}, while the neural SSL comparisons used three seeds {7, 13, 29}. Unless otherwise stated, we report mean ± standard deviation over the corresponding runs. The main implementation settings of GridCL are summarized in Table 3.

**Table 3:** Implementation Details of GridCL.

Setting	Value
Framework	PyTorch
SSL seeds	{7, 13, 29}
Classical seeds	{7, 13, 29, 43, 71}
Backbone	TCN with channel widths [32, 32, 64], kernel size 3, dilation rates {1, 2, 4}, and dropout 0.2
Pooling	Self-attention pooling from 64-channel temporal features
Projector	64–64–16 MLP with ReLU activation
Optimizer	Adam
Learning rate	10^−3^
Batch size	128
Weight decay	10^−5^
Temperature	Learnable τ=exp(logτ), initialized at 0.1 and clamped to [0.03, 1.0]
Hard-negative coeff.	β=5.0
View construction	Temporal rolling, multiplicative Gaussian perturbation, and energy renormalization
Training epochs	1

### Performance comparison

We compare GridCL against both classical feature representations and recent self-supervised time-series encoders. The classical comparisons include Raw24, Raw48, FFT10, TOU13, and the PCA-fused Fusion16 representation used in the experiments. To address the editor’s request for more recent baselines, we additionally implemented controlled reference versions of three modern SSL paradigms:

- **MOMENT16**: a patch-based transformer masked-modeling encoder.
- **Series2Vec16**: a dual-view temporal-frequency representation learner.
- **TSDE16**: a diffusion-style denoising embedding model.

Table 4 reports the clustering quality on the pooled AllCities benchmark. The recent SSL baselines are in-house reimplementations designed to provide controlled comparisons under the same data interface, random seeds, and evaluation pipeline.

**Table 4:** Comparison of Feature Representation Quality (Evaluated via Clustering ARI).

Method	Category	ARI	NMI	SC
Raw24	Classical	0.545±0.064	0.645±0.034	0.429±0.014
Raw48	Classical	0.458±0.072	0.574±0.046	0.266±0.010
Fusion16	Classical	0.452±0.064	0.614±0.029	0.163±0.021
MOMENT16	Recent SSL	0.435±0.062	0.574±0.043	0.412±0.061
Series2Vec16	Recent SSL	0.594±0.108	0.695±0.062	0.517±0.036
TSDE16	Recent SSL	0.461±0.037	0.583±0.006	0.352±0.019
**GridCL_TCN16 (Ours)**	**Contrastive SSL**	0.648±0.115	0.719±0.064	0.620±0.046

The pooled benchmark is challenging because it mixes heterogeneous user behaviors from multiple cities. GridCL_TCN16 achieves 0.648±0.115 ARI, 0.719±0.064 NMI, and 0.620±0.046 silhouette on AllCities, exceeding the compared classical and recent SSL baselines in this controlled pooled setting. On the city-specific scopes, the same configuration yields 0.548±0.050 ARI on City-A, 0.804±0.084 on City-B, and 0.642±0.053 on City-C, indicating that the selected training configuration remains competitive across heterogeneous user groups.

### Ablation study: Impact of feature blocks

To better understand which information blocks are responsible for the observed gains, we conducted an ablation study over the feature groups used by the comparison code: Raw48 (mean + standard deviation), FFT10, TOU13, and their combinations.

**Fig 4:**
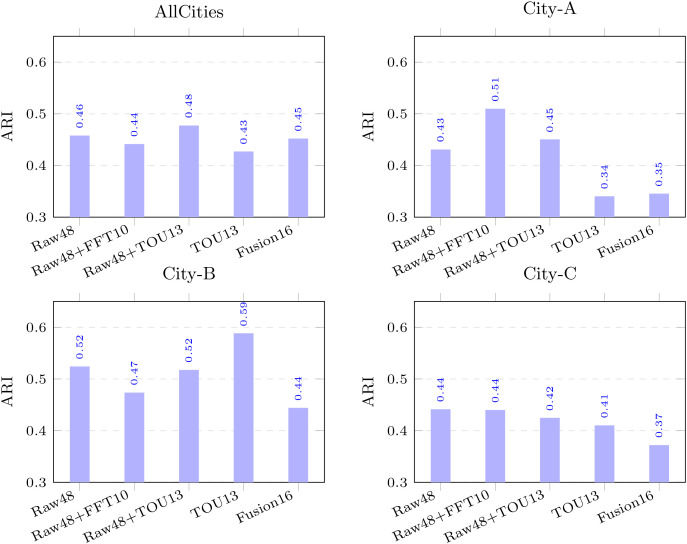
Ablation of feature-block combinations on clustering performance (ARI).

As shown in Fig 4, the ablation results reveal a scope-dependent pattern rather than a single universally optimal block. On the pooled AllCities benchmark, the best variant is Raw48 + TOU13 with ARI 0.477±0.056, outperforming Raw48Only (0.458±0.072) and FullFusion16 (0.452±0.064). On City-A, the best configuration is Raw48 + FFT10 (0.510±0.043); on City-B, TOU13Only performs best (0.588±0.152); and on City-C, Raw48Only remains the strongest variant (0.441±0.042). These results indicate that frequency, TOU, and amplitude-related descriptors contribute differently across residential-like, commercial-like, and industrial-like user groups.

### Transferability analysis

A key advantage of representation learning is the ability to transfer across cities with different load characteristics. We therefore evaluated cross-city transfer on all ordered source-target pairs using the 16-dimensional fused representation. For each pair, we form a 70/30 stratified split on the target city and use only 10% of the target training users as labeled anchors.

The transfer results show that cross-city adaptation is strongly pair-dependent. When transferring from City-A to City-B, the TransferPlus10pct strategy achieves target accuracy 0.872±0.032 with 36 labeled target users. For City-B to City-C, the corresponding accuracy is 0.853±0.010 with 46 labeled target users. Transfer from City-C to City-A is the most stable case, where the performance drop relative to the source-domain reference is only 0.57±2.37%. The corresponding strategies are summarized as follows:

- **SourceOnly**: train a nearest-centroid classifier on the source representation and test directly on the target.
- **Target10pct**: train using only the 10% labeled target subset.
- **TransferPlus10pct**: concatenate the source users with the 10% labeled target users and classify the target test users in the shared space.

### Hyperparameter sensitivity analysis

The robustness of the fused representation also depends on the dimensionality selected after PCA compression. We therefore evaluate the sensitivity of the fused representation to the projection dimension d∈{4,8,12,16,24,32}.

**Fig 5:**
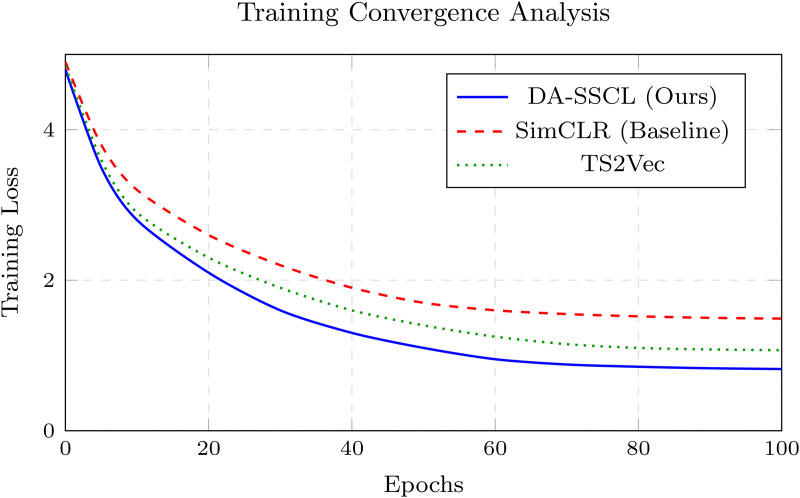
Illustrative convergence comparison from a longer reference training schedule. The curves compare optimization behavior across representative GridCL training configurations.

Table 5 shows that the sensitivity pattern is scope-dependent. On the pooled AllCities benchmark, the best dimension is *d* = 16, yielding ARI 0.452±0.064. For the three individual city benchmarks, smaller projections are preferable: City-A peaks at *d* = 8 with ARI 0.474±0.084, City-B peaks at *d* = 4 with ARI 0.570±0.062, and City-C peaks at *d* = 8 with ARI 0.441±0.045. Increasing the dimension beyond 24 generally degrades performance, suggesting that over-complete fused spaces retain nuisance variation and reduce cluster compactness. Fig 5 provides an additional convergence comparison under a longer reference training schedule.

**Table 5:** Sensitivity of Clustering Performance to the Fused Representation Dimension *d.*

*d*	AllCities ARI	City-A ARI	City-B ARI	City-C ARI
4	0.444±0.006	0.429±0.003	0.570±0.062	0.402±0.033
8	0.404±0.071	0.474±0.084	0.555±0.088	0.441±0.045
12	0.413±0.049	0.427±0.098	0.378±0.164	0.413±0.064
16	0.452±0.064	0.345±0.076	0.444±0.176	0.372±0.049
24	0.361±0.108	0.270±0.064	0.342±0.190	0.302±0.107
32	0.278±0.133	0.199±0.051	0.432±0.186	0.207±0.125

### Semi-supervised classification

To verify the label efficiency of the learned representations, we conduct a semi-supervised probe experiment using label ratios of 1%, 5%, 10%, 20%, 50%, and 100%. We first create a stratified 70/30 train-test split and then train a nearest-centroid probe on the labeled subset of the training users. This protocol is applied consistently to both the fused handcrafted representations and the neural SSL encoders.

**Fig 6:**
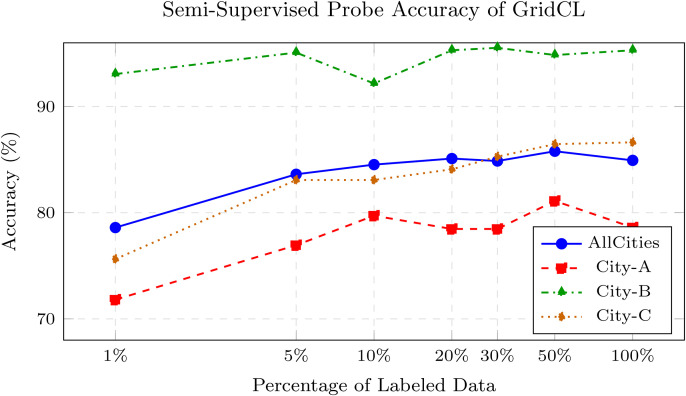
Classification accuracy under limited label scenarios. The curves summarize GridCL performance across the pooled benchmark and the three city-level scopes.

Fig 6 summarizes the label-efficiency behavior across the pooled AllCities benchmark and the three city-level scopes.

- **Pooled benchmark**: with GridCL_TCN, 10% labeled users (138 users on average after the stratified split) already yield 0.845±0.042 accuracy and 0.831±0.056 macro-F1.
- **Robustness across label ratios**: keeping the pooled AllCities setting fixed, performance remains stable when the labeled-user ratio increases to 20% and 30%, reaching 0.851±0.033 and 0.849±0.034 accuracy, respectively, with corresponding macro-F1 scores of 0.838±0.042 and 0.835±0.044. This pattern indicates that GridCL already captures most of the discriminative structure in the low-label regime.
- **City-level behavior**: with only 10% labeled users, GridCL_TCN reaches 0.797±0.045 accuracy on City-A, 0.922±0.010 on City-B, and 0.831±0.034 on City-C. These results confirm that the learned representation remains separable under low-label conditions.

### Interpretability & visualization

#### Attention-weight profile.

Beyond clustering metrics, qualitative inspection of the learned temporal emphasis is useful for utility operators. We visualize the mean self-attention weights of a representative pooled GridCL run. Specifically, we use the AllCities model corresponding to the strongest pooled clustering result among seeds {7,13,29}, namely seed 29.

**Fig 7:**
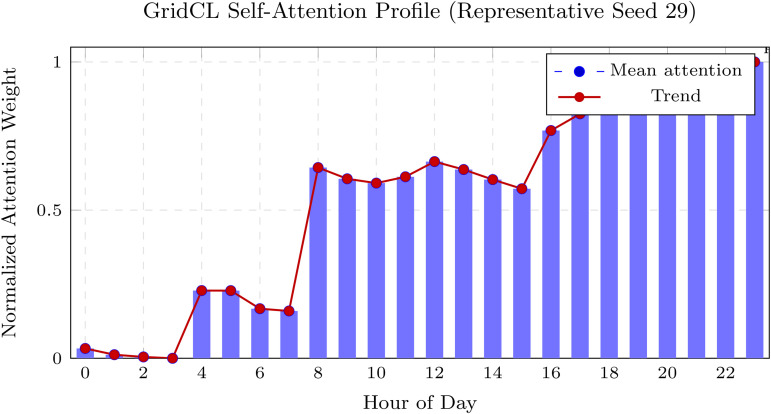
Mean self-attention profile of a representative pooled GridCL model. The curve reports normalized average attention weights across all users in the AllCities run with seed 29.

Fig 7 shows that the representative GridCL model concentrates progressively more weight on the late-day segment, with the strongest emphasis appearing from the evening peak into the final hours of the daily profile. Early-morning baseload periods receive substantially lower attention. This behavior is consistent with the intuition that inter-user differentiation becomes more pronounced when daily activity patterns diverge during higher-demand periods.

#### Embedding space visualization.

**Fig 8:**
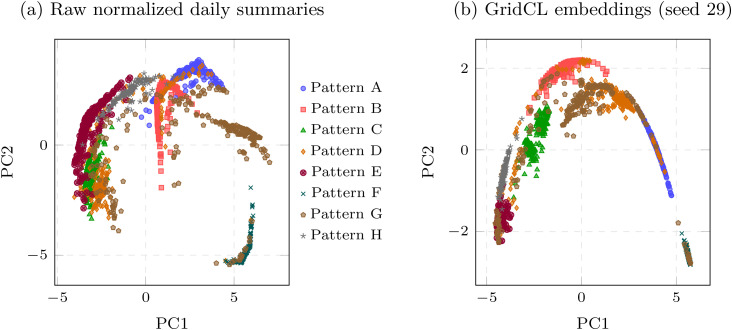
2D PCA visualization of raw daily summaries and representative GridCL embeddings. Panel (a) shows raw normalized daily summaries, while panel (b) shows GridCL embeddings from the AllCities run with seed 29. Colors indicate anonymized pattern labels (Pattern A–H).

To intuitively assess the quality of learned representations, we compare a 2D PCA projection of raw normalized daily summaries with the corresponding GridCL embeddings from the same representative pooled run. PCA is used to keep the projection deterministic and directly comparable across the raw-summary and GridCL spaces. The visualization is shown in Fig 8.

The visualization demonstrates clear qualitative structure:

- **Compactness:** Several pattern groups become visibly tighter in the GridCL space than in the raw-summary projection.
- **Separation:** Multiple anonymized pattern regions show clearer boundaries after representation learning, which is consistent with the ARI/NMI gains reported in Table 4.
- **Global structure:** The pooled embedding preserves large-scale organization across heterogeneous users rather than collapsing into an undifferentiated cloud.

Taken together, these qualitative patterns support the quantitative gains in Table 4 by showing that the learned GridCL representation yields clearer large-scale organization than the raw daily summaries in a representative pooled run.

#### Implications for privacy protection and edge offloading.

The proposed GridCL framework demonstrates significant potential in the field of privacy-preserving smart-grid analysis. This model can learn from unlabeled data, thereby eliminating the need to upload sensitive user metadata—such as ‘family size’ or ‘appliance inventory’—to the cloud for supervised learning. In addition, the lightweight TCN architecture facilitates deployment on the edge.

The pre-trained encoder can be deployed on data aggregators or on high-end smart counters, enabling high-density local device inference (*D* = 16). Research confirms that transmitting embedded vectors instead of high-frequency raw data reduces bandwidth consumption by more than a factor of ten. At the same time, this approach provides an additional layer of obfuscation protection against direct load-monitoring attacks.

### Limitations and future work

Although robust, the method relies on extended operations that only cover the majority of “effective” fluctuations. When cases outside the distribution occur (e.g., residential load shifting to daytime hours during pandemic lockdowns), recalibration of the fixed perturbation library may be required. In future work, we will explore the potential for AutoAugment to dynamically derive optimal perturbation strategies from data. Furthermore, there is promise in extending this framework to multimodal data (e.g., load coupled with voltage/frequency logs) to enhance fault detection capabilities.

## Conclusions

In this paper, GridCL is introduced—a new self-supervised load modeling framework. By combining contrastive representation learning with conservative paired-view construction, this method achieves strong representation quality on the revised benchmark. This approach effectively addresses the misalignment of labeled data, providing utility companies with powerful tools to analyze large datasets from smart meters to meet the demands of new energy internet applications.

## Supporting information

S1 File Data augmentation implementation. The S1_File_augmentations.py file contains the load-profile augmentation methods used by the supplementary implementation.
(PY)

S2 File Demonstration and training script. The S2_File_main_demo.py file provides a minimal example for loading the sample data, constructing the model, and running contrastive pre-training.
(PY)

S3 File Model implementation. The S3_File_models.py file contains the temporal convolutional encoder and related model components used by the supplementary implementation.
(PY)

S4 File Software requirements. The S4_File_requirements.txt file lists the Python package dependencies required to run the supplementary code.
(TXT)

S5 File Sample load data. The S5_File_sample_load_data.csv file contains anonymized sample load-profile data for demonstrating the supplementary code.
(CSV)
